# A dual graph neural network for drug–drug interactions prediction based on molecular structure and interactions

**DOI:** 10.1371/journal.pcbi.1010812

**Published:** 2023-01-26

**Authors:** Mei Ma, Xiujuan Lei

**Affiliations:** 1 School of Computer Science, Shaanxi Normal University, Xi’an, China; 2 School of Mathematics and Statistics, Qinghai Normal University, Qinghai, China; Tel Aviv University, ISRAEL

## Abstract

Expressive molecular representation plays critical roles in researching drug design, while effective methods are beneficial to learning molecular representations and solving related problems in drug discovery, especially for drug-drug interactions (DDIs) prediction. Recently, a lot of work has been put forward using graph neural networks (GNNs) to forecast DDIs and learn molecular representations. However, under the current GNNs structure, the majority of approaches learn drug molecular representation from one-dimensional string or two-dimensional molecular graph structure, while the interaction information between chemical substructure remains rarely explored, and it is neglected to identify key substructures that contribute significantly to the DDIs prediction. Therefore, we proposed a dual graph neural network named DGNN-DDI to learn drug molecular features by using molecular structure and interactions. Specifically, we first designed a directed message passing neural network with substructure attention mechanism (SA-DMPNN) to adaptively extract substructures. Second, in order to improve the final features, we separated the drug-drug interactions into pairwise interactions between each drug’s unique substructures. Then, the features are adopted to predict interaction probability of a DDI tuple. We evaluated DGNN–DDI on real-world dataset. Compared to state-of-the-art methods, the model improved DDIs prediction performance. We also conducted case study on existing drugs aiming to predict drug combinations that may be effective for the novel coronavirus disease 2019 (COVID-19). Moreover, the visual interpretation results proved that the DGNN-DDI was sensitive to the structure information of drugs and able to detect the key substructures for DDIs. These advantages demonstrated that the proposed method enhanced the performance and interpretation capability of DDI prediction modeling.

## Author summary

Drug-drug interactions (DDIs) may cause adverse effects that damage the body. Therefore, it is critical to predict potential drug-drug interactions. The majority of the prediction techniques still rely on the similarity hypothesis for drugs, sometimes neglect the molecular structure, and fail to include the interaction information between chemical substructure when predicting DDIs. We exploited this idea to develop and confirm the role that molecular structure and interaction information between chemical substructure play in DDIs prediction. The model includes a molecular substructure extraction framework to explain why substructures contribute differently to DDIs prediction, and a co-attention mechanism to explain why the interaction information between chemical substructure can improve DDIs prediction. Compared to state-of-the-art methods, the model improved the performance of DDIs prediction on real-world dataset. Furthermore, it could identify crucial components of treatment combinations that might be efficient against the emerging coronavirus disease 2019 (COVID-19).

This is a *PLOS Computational Biology* Methods paper.

## Introduction

With the rapid development of machine learning techniques, many AI technologies have been successfully applied in a variety of tasks for drug discovery, such as drug-drug interactions (DDIs) [1]. One of the main issues in these studies is how to learn expressive representation from molecular structure [2]. Most of the conventional molecular representation are based on hand-crafted features and heavily rely on time-consuming biological experimentations [3].

The most common molecular representation method called simplified molecular input line entry specification(SMILES), is the molecular linear notation that encodes the molecular topology on the basis of chemical rules [4,5], while this line of work suffered from insufficient labeled data for specific molecular tasks. More recently, among the promising deep learning architectures, graph neural networks (GNNs) have gradually emerged as a powerful candidate for modeling molecular data [6–8]. A molecule is naturally treated as a graph based on its geometry, where an atom serves as the node and a chemical bond serves as the edge. Therefore, a molecule is normally mapped to an undirected graph and defined as *G* = (*V*, *E*), where *V* and *E* are the sets of all atoms and chemical bonds in the molecule, respectively. Moreover, to better encode the interactions between atoms, a message passing neural network named MPNN was designed to utilize the attributed features of both atoms and edges [9]. It is a general framework for learning node embeddings or learning the entire graph representations. The MPNN used the basic molecular graph topology to obtain structural information through neighborhood feature aggregation and pooling methods [10,11].

DDIs prediction is one of the applications of molecular representation [12–14]. DDIs is referred to as a situation where the pleasant or adverse effects caused by the co-administration of two drugs, which may cause adverse drug events and side effects that damage the body [15,16]. In order to avoid such events, it’s urgent to develop computational approaches to detect DDIs [17].

Various machine learning methods have been proposed and have greatly contributed to the DDIs prediction [18–20]. The vast majority of these techniques rely on the drug similarity assumptions, where it is believed that if drugs A and B interact to produce a specific biological effect, then drugs similar to drug A (or drug B) are likely to interact with drug B (or drug A) to produce the same effect. Drugs are, therefore, processed depending on their similarities in chemical structures; as well as additionally, in other features such as their individual side effects, targets, pathways [21,22].

Recently, many deep learning methods have been developed and have shown encouraging performance in DDIs prediction tasks [23–26]. For instance, Deng et al. [24] proposed a multi-modal deep learning framework combined diverse drug features to predict DDIs. Feng et al. [25] applied deep graph auto-encoder to learn latent drugs representations fed to a deep feedforward neural network for DDIs prediction. Liu et al. [27] introduced a deep attention neural network framework for drug-drug interaction prediction, which can effectively integrate multiple drug features. For adverse drug-drug interaction (ADDI), Zhu et al. [28] employed eight attributes and developed a discriminative learning algorithm to learn attribute representations of each adverse drug pair for exploiting their consensus and complementary information in multi-attribute ADDI prediction. And then they designed three dependence guided terms among molecular structure, side effect and ADDI to guide feature selection and put forward a discriminative feature selection model DGDFS for ADDI prediction [29]. Because combination therapy can boost efficacy and reduce toxicity, recent approaches have used deep learning to identify synergistic drug combinations for the new coronavirus disease 2019 (COVID-19) [30–32]. Jin et al. [31] presented a new deep learning architecture ComboNet for predicting synergistic drug combinations for COVID-19. Howell et al. [33] developed a computational model of SARS-CoV-2-host interactions used to predict effective drug combinations.

Although these methods achieved inspiring results, there are still mostly unexplored in DDIs prediction tasks especially as far as feature extraction from the raw representations (i.e., chemical structures) of drugs are concerned. Most of the existing methods predict DDIs relying on the similarity assumption of drugs or on manually engineered features [34,35]. However, molecular structure-based methods regard drugs as independent entities, and predict DDIs only by relying on drug pairs. This is no need for external biomedical knowledge. It has been proven that DDIs usually depend only on a few substructures as a whole [36,37]. SSI-DDI [34] and GMPNN-CS [35], two recent methods that both leveraged the powerful feature extraction ability of deep learning, work directly on raw molecular chemical structures of drugs using GNNs. SSI-DDI used graph attention (GAT) layers [38] to learn a comprehensive feature representation of a drug from substructures, while GMPNN-CS introduced gated message passing neural network which learns chemical substructures with different sizes and shapes from the molecular graph representations of drugs for DDIs prediction. However, the gates are computed before the message passing, which means that they did not fully exploit the molecular structure information.

In this study, we proposed a DDIs prediction approach called DGNN-DDI that uses dual GNN to extract drug feature representation while taking into account drug substructure and the interaction information between chemical substructure. To extract the molecular substructures features, we first constructed a directed message passing neural network with substructure attention mechanism (SA-DMPNN) by fully considering the flexible-sized and irregular-shaped of drug molecules substructures. Second, we used co-attention mechanism [39] to determine the importance weight by learning the interaction scores between the substructures features of the two drugs. After that, we concatenated the weighted substructures features for each drug to obtain the final feature, which was used to predict the potential interaction probability of the existing drugs and drugs. We evaluated our model using real-world dataset, and the experiments demonstrated that our DGNN-DDI is superior in predicting the potential DDIs. The method was applied to predict anti-COVID-19 drug combinations. The main contributions of this work are summarized as the following:

1. DGNN-DDI takes into account the key molecular substructure feature of the drugs, which is conducive to learning high-quality features.
2. DGNN-DDI leverages the interaction information between chemical substructure to identify substructures with interactions, which can enhance the final feature of drug and also contribute to improving the prediction accuracy of DDIs.
3. The method is applied to predict anti-COVID-19 drug combinations using real-world dataset.

## Results and discussion

### Dataset

We evaluated the model performance in DrugBank [40], which is a unique bioinformatics and cheminformatics resource that combines detailed drug data with comprehensive drug target information. The dataset contains 1706 drugs and 191808 DDIs tuples classified into 86 interaction types, which describes how one drug affects the metabolism of another one. Each drug is associated with their SMILES and we converted it into molecular graph using RDKit. In the dataset, each drug pair is only associated with a single type of interaction.

### Experiment setting

In our study, we split the dataset randomly into training (60%), validation (20%), and test (20%) for the DDIs prediction task. The message passing steps *T* was searched from {1, 2, 3, 4, 5}, and the Multi-GNN layers *L* was searched from {1, 2, 3, 4,5}. Because of the model was dual, *T* and *L* was determined to be 3 through subsequent parameter analysis. After parameter analysis, we considered the following hyper-parameter settings. Dimension of *h*_*i*_ in Eq 12 was searched from {32, 64, 128}. The model was trained on mini-batches DDI tuples tuned from {128, 256, 512} using the Adam optimizer [41] with a learning rate *lr* tuned from {1e-2, 1e-3, 1e-4}. Additionally, an exponentially decaying scheduler of 0.96*e*(where *e* is the current epoch) was set on the learning rate. We discovered that the best performance was obtained when the *T* = *L* = 3, *h*_*i*_ ∈ *R*^64^, *lr* = 1*e* − 4 and batch size was 256. The number of epochs was 50. To avoid overfitting, we applied a weight decay of 5 × 10^−4^ for all methods. Like most of the literatures [42,43], we trained the comparison methods with the same parameter settings as DGNN-DDI, including learning rate, optimizer, batch size, weight decay, hidden dimension and number of epochs. But the message passing steps *T* or layers *L* was taken from original manuscript. The performance metrices included accuracy (ACC), area under the curve (AUC), F1-score (F1), precision (Prec), recall (Rec) and area under the precision and recall curve (AUPR).

### Comparative analysis with other methods

On the DrugBank dataset, we compared the proposed model with cutting-edge approaches to verify its efficacy. Only chemical structural information is taken into account by these approaches as an input, and combined drug-drug information is integrated in some way during the learning process.

- SA-DDI [44]: a GNN that used a message-passing neural network and a substructure-substructure interaction module to learn thorough and useful features. SA-DDI extracted features with message passing step *T* = 10 for DDIs prediction.
- SSI-DDI [34]: considered each node hidden features as sub-structures and then computes interactions between these substructures to determine the final DDI prediction. SSI-DDI stacked *L* = 4 layers of graph attention (GAT) for DDIs prediction.
- GMPNN-CS [35]: a GNN architecture that introduced gates message passing mechanism to control the flow of message passing of GNN. GMPNN-CS learned chemical substructures with message passing step *T* = 10 for DDIs prediction.
- GAT-DDI [35]: replaced the GNN architecture in GMPNN-CS with GAT for drug representations, which are directly used for DDI prediction. GAT-DDI learned chemical substructures with message passing step *T* = 10 for DDIs prediction.

Table 1 summarizes metric scores of all prediction models, and results demonstrate that DGNN-DDI outperforms other methods on all metric scores for the DrugBank dataset, which show the effectiveness of the proposed DGNN-DDI for DDI prediction.

**Fig 1:**
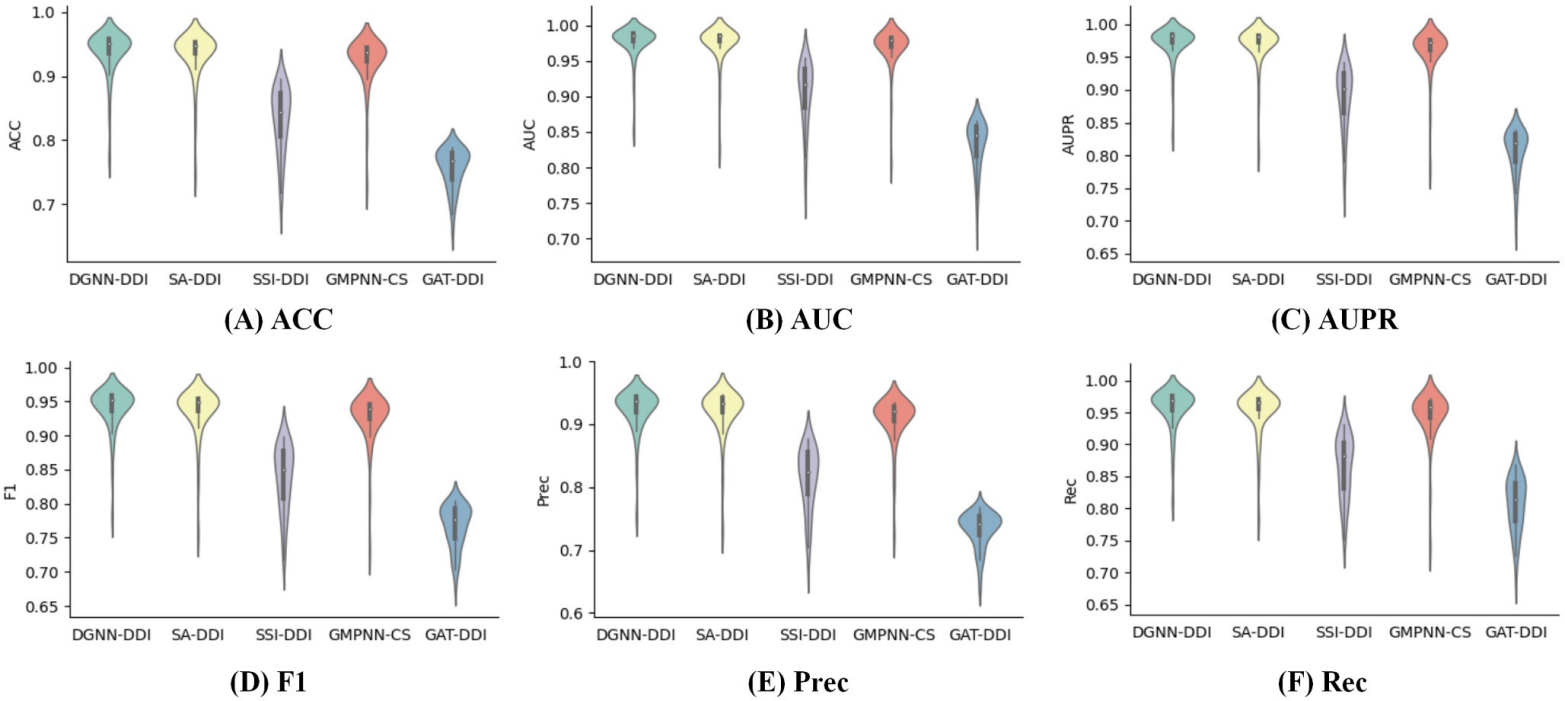
Violin plots displaying metric scores of all models.

To further analyze the performances of prediction models, we used Fig 1A–1F to display all metric scores of different methods. These violin plots clearly show that DGNN-DDI produces better performances for these metrices than the competing methods.

**Table 1:** Comparison results in % of the proposed DGNN-DDI and baselines on the dataset.

	ACC	AUC	F1	Prec	Rec	AUPR
GAT-DDI	0.7894	0.8653	0.8045	0.7676	0.8682	0.8398
GMPNN-CS	0.9485	0.9834	0.9495	0.9346	0.9725	0.9785
SA-DDI	0.9565	0.9868	0.9573	**0.9472**	0.9746	0.9834
SSI-DDI	0.8965	0.9541	0.8993	0.8763	0.9321	0.9420
DGNN-DDI	**0.9609**	**0.9894**	**0.9616**	**0.9472**	**0.9788**	**0.9863**

**Fig 2:**
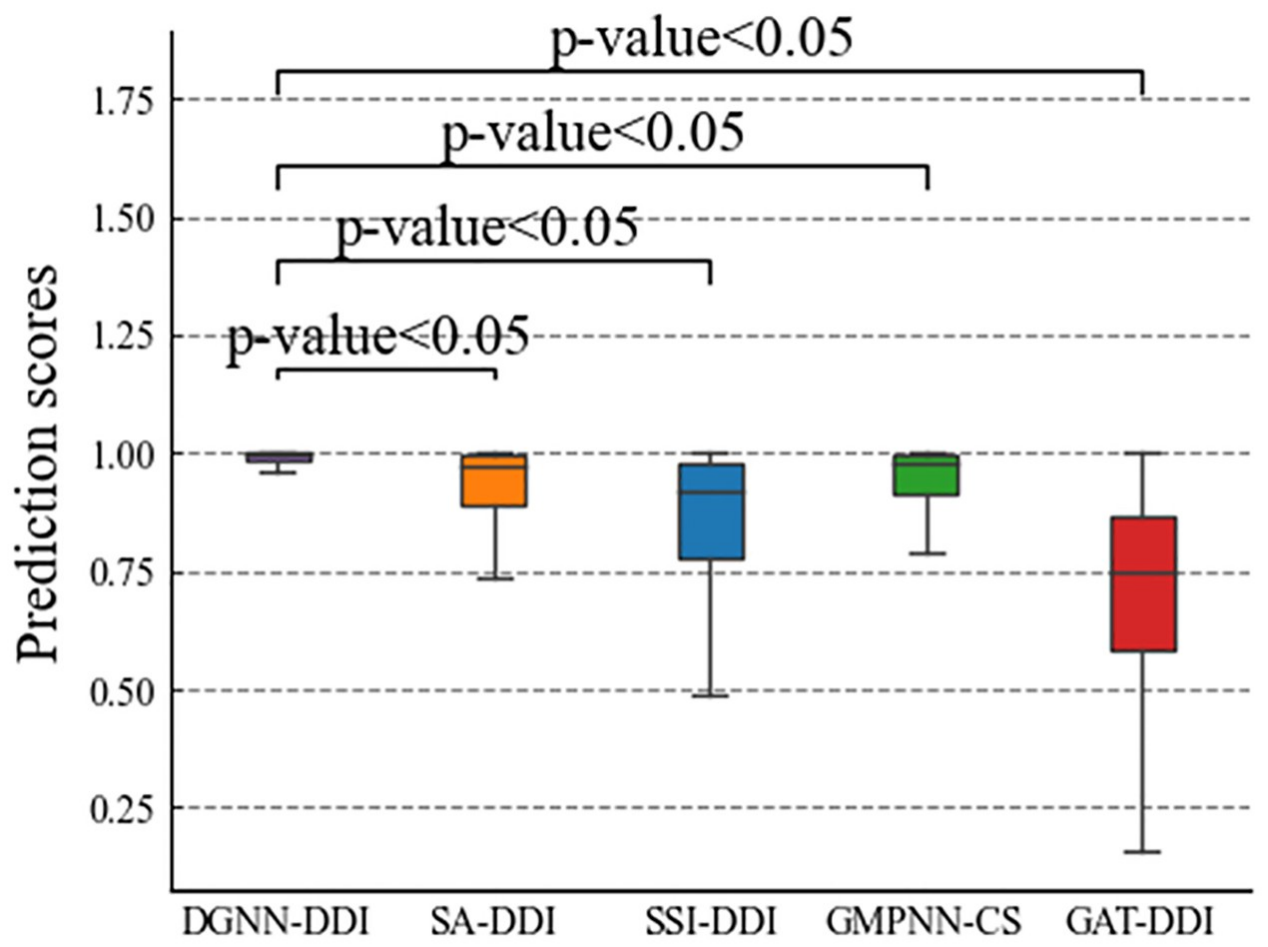
The significant difference between DGNN-DDI and other models in terms of predicted scores.

Moreover, we conducted a statistical analysis to test the differences between DGNN-DDI and other state-of-the-art methods. We conducted statistical significance tests by using predicted scores, and paired *t*-test results are demonstrated in Fig 2. For the DDI prediction, the proposed method DGNN-DDI significantly (*p*-value < 0.05) improves the performances compared to other state-of-the- art methods.

**Fig 3:**
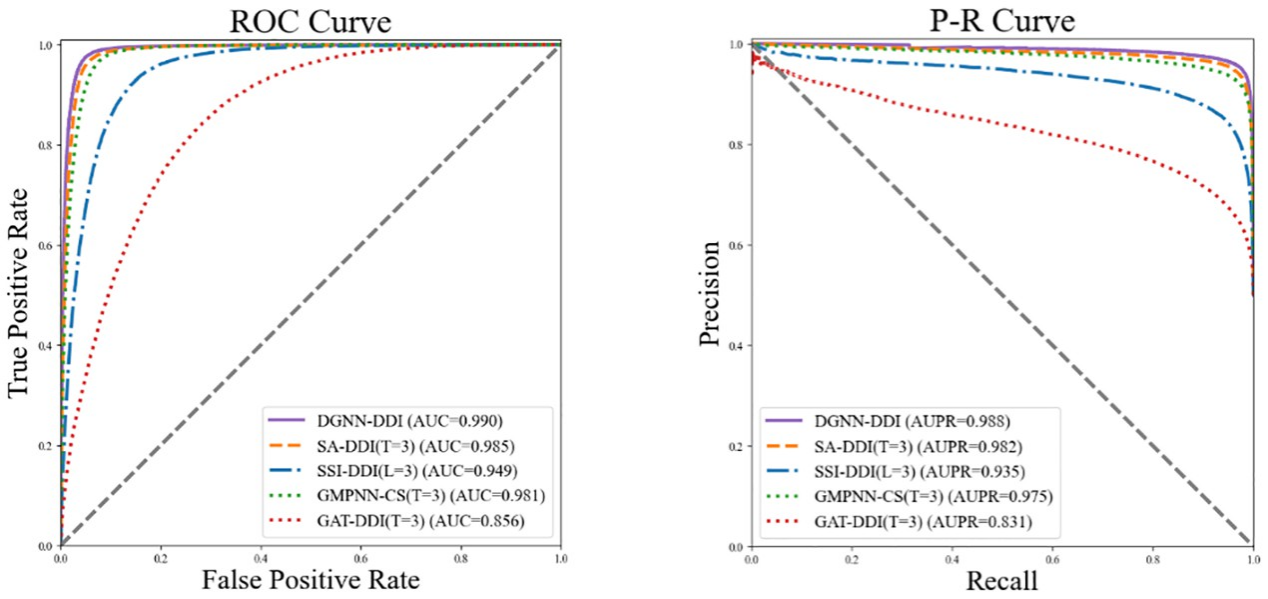
The ROC curves and P-R curves of all models, where *T* = 3 or *L* = 3 for all models.

To further demonstrate the superiority of GNN-DDI, we set *T* = 3 or *L* = 3 for all comparison methods, which is consistent with DGNN-DDI. Fig 3 displays the ROC curves (receiver operating characteristic curves) and P-R curves (precision-recall curves) of all models. Clearly, DGNN-DDI performs best among all methods, demonstrating once more its strong potential for DDIs prediction.

### Parameter analysis

**Fig 4:**
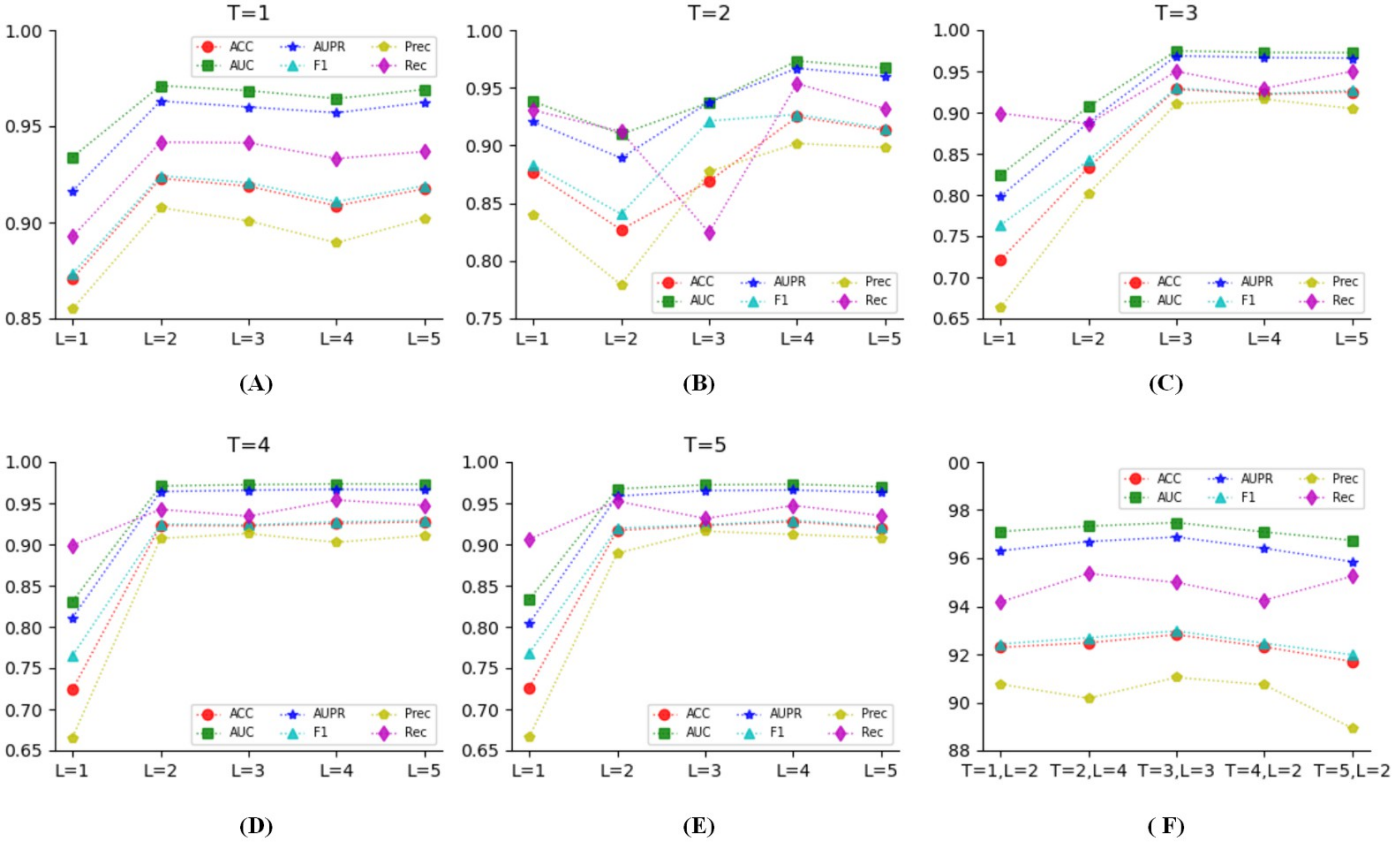
The effects of steps *T* and layers *L*.

The parameters *T* and *L* have a significant impact on the extraction of substructures with variable sizes and forms during the processing of molecular features learning. We tested all 25 combinations of steps *T* and layers *L*, as shown in Fig 4. The distribution of all metric scores under all 25 combinations are shown in Fig 4A–4E, respectively. It can be seen that when *T* = 1, *L* = 2; *T* = 2, *L* = 4; *T* = *L* = 3; *T* = 4, *L* = 2; *T* = 5, *L* = 2 all metric scores are superior to other combinations. We further compared these five combinations, as shown in Fig 4F, when *T* = *L* = 3 shows a better performance than other combinations.

**Fig 5:**
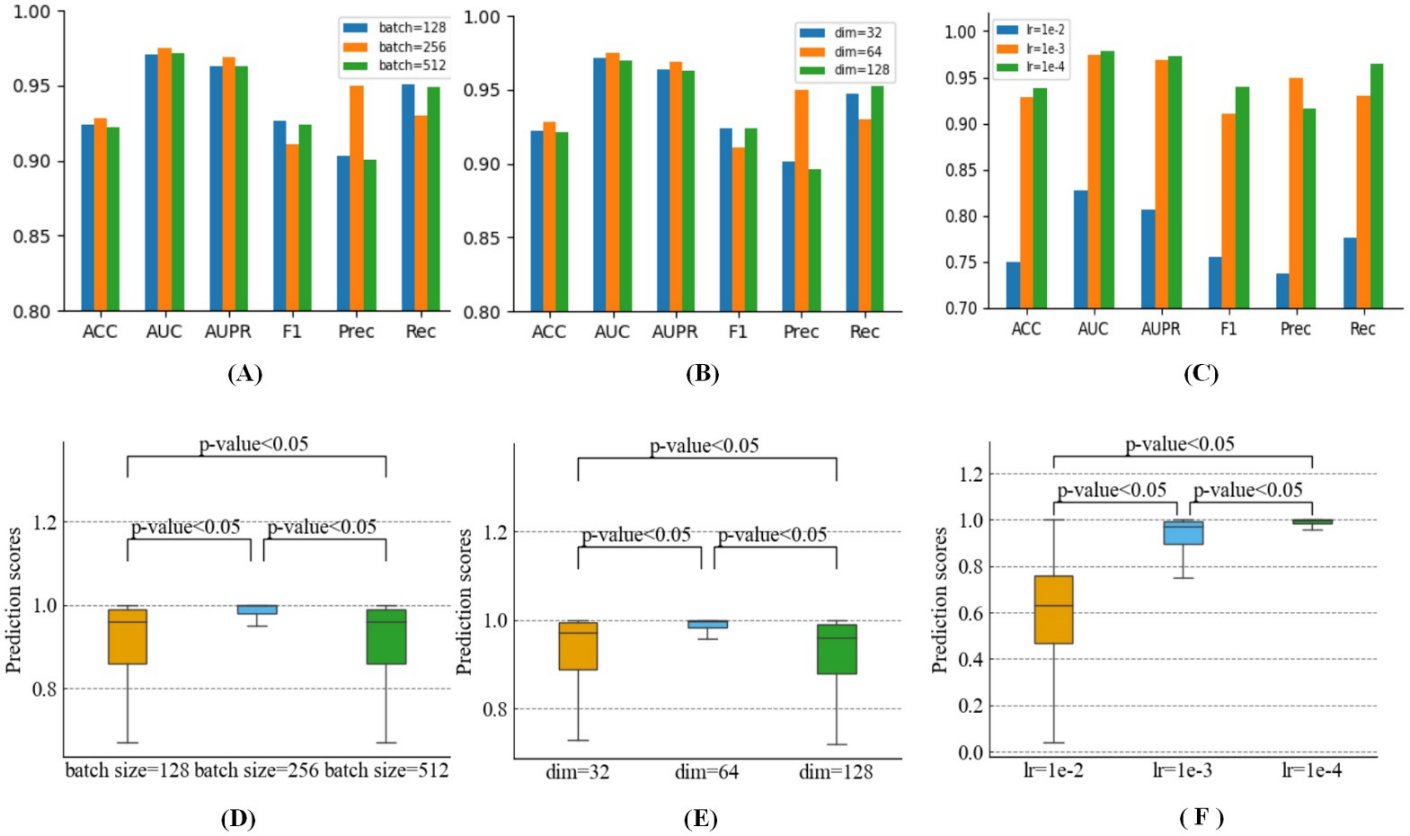
Parametric analysis. (A) Effects of batch size. (B) Effect of hidden dimension. (C) Effects of learning rate. (D) The significance analysis of batch size in terms of predicted scores. (E) The significance analysis of hidden dimension in terms of predicted scores. (F) The significance analysis of learning rate in terms of predicted scores.

The size of the batch is particularly significant since the DGNN-DDI is sampled and trained in batches. It will be challenging to converge if the batch size is too small. While if the batch is too large, a large amount of computation is required. As shown in Fig 5A, we investigated the impact of various batch sizes on the methodology. The method has the best performance when the batch size is equal to 256. As demonstrated in Fig 5B and 5C, we also looked into how hidden dimensions and learning rates affected the performance of the model. Moreover, we conducted a significance analysis to test the differences on different values of batch size, learning rate and hidden dimension, respectively. Using predicted score, we conducted statistical significance tests, the paired *t*-test results are demonstrated in Fig 5D, 5E and 5F. For the three parameters, when the *h*_*i*_ ∈ *R*^64^, *lr* = 1*e* − 4 and batch size is equal to 256, DGNN-DDI significantly (*p*-value < 0.05) improves the performances compared to other values.

### Ablation study

In our designs, the successful construction of the DGNN-DDI highly relies on D-MPNN with substructure attention mechanism (SA-DMPNN) and interaction-aware substructure extraction (Multi-GNN). The substruction attention is used to extract substructures with arbitrary size and shape. The relevance of substructure interactions with co-attention is expected to enhance the model performance to the final DDI prediction. We conducted experiments by removing the substructure-attention mechanism (SA) and/or co-attention layer (CA). Table 2 summarizes the contributions of SA and CA. The results show that both SA and CA are necessary for DGNN-DDI.

**Table 2:** Investigating the contributions of substructure-attention mechanism and co-attention layer.

	ACC	AUC	F1	Prec	Rec	AUPR
DGNN-DDI_no_SA	0.9072	0.9482	0.8979	0.8833	0.9155	0.9248
DGNN-DDI_no_CA	0.8882	0.9461	0.8915	0.8755	0.9413	0.9313
DGNNDDI_no_SA_CA	0.8858	0.9445	0.8898	0.8785	0.9308	0.9273
DGNN-DDI	**0.9609**	**0.9894**	**0.9616**	**0.9472**	**0.9788**	**0.9863**

**Fig 6:**
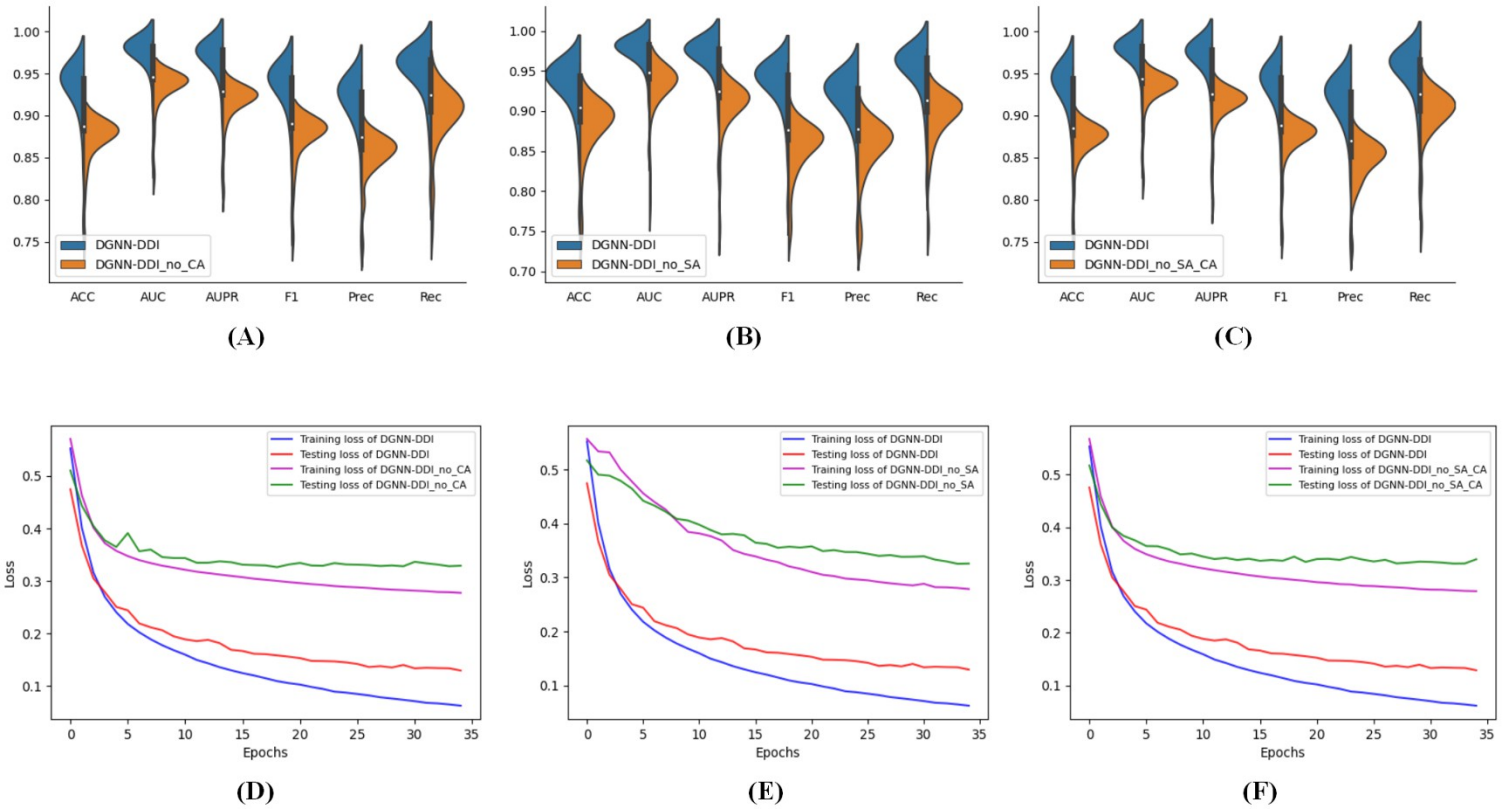
Analysis of the substructure attention mechanism (SA) and co-attention layer (CA). (A)-(C) The metric scores of DGNN-DDI and without SA and/or CA. (D)-(F) The training and testing losses for DGNN-DDI and without SA and/or CA.

Fig 6A–6C also shows that the model performs poorly without SA, CA, or SA and CA, showing the necessity of SA and CA. Furthermore, we observed that the results decrease greatly when applying either SA or CA. However, the improvement of using both is larger than the only one, highlighting the effectiveness of DGNN-DDI. Additionally, as demonstrated in Fig 6D–6F, SA and CA can expedite training while also enhancing generalization ability.

### Visual explanations for DGNN-DDI

**Fig 7:**
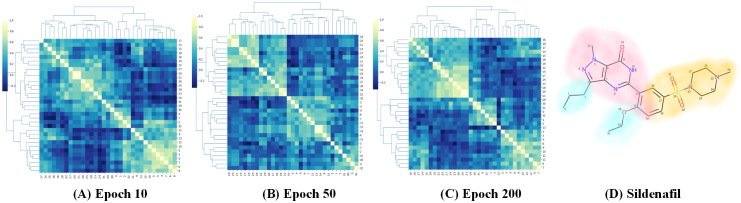
Heat maps of the atom similarity matrix for drug sildenafil.

**Fig 8:**
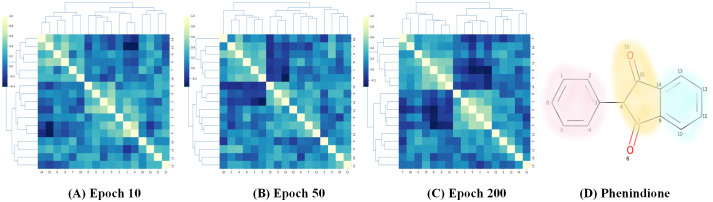
Heat maps of the atom similarity matrix for drug phenindione.

We conducted visual explanation-related experiments to rationalize the DGNN-DDI. To investigate how the atom hidden vectors evolved during the learning process, we obtained the similarity coefficient between atom pairs by measuring the Pearson correlation coefficient for those hidden vectors. We chose the hidden vectors after the last iteration (i.e., *h*_*i*_ in Eq 12). Figs 7 and 8 give two drugs with their atom similarity matrices during the learning process. The cluster heat maps show some degree of chaos at the beginning and then clearly group into clusters during the learning process where the corresponding substructures for clusters are highlighted in the drugs. Taking Fig 7 as an example, we found that the atoms in sildenafil at epoch 50 approximately separate into three clusters. This finding is in accordance with our intuition regarding the sildenafil structure.

These results suggest that the DGNN-DDI can capture the structure information of a molecule.

**Fig 9:**
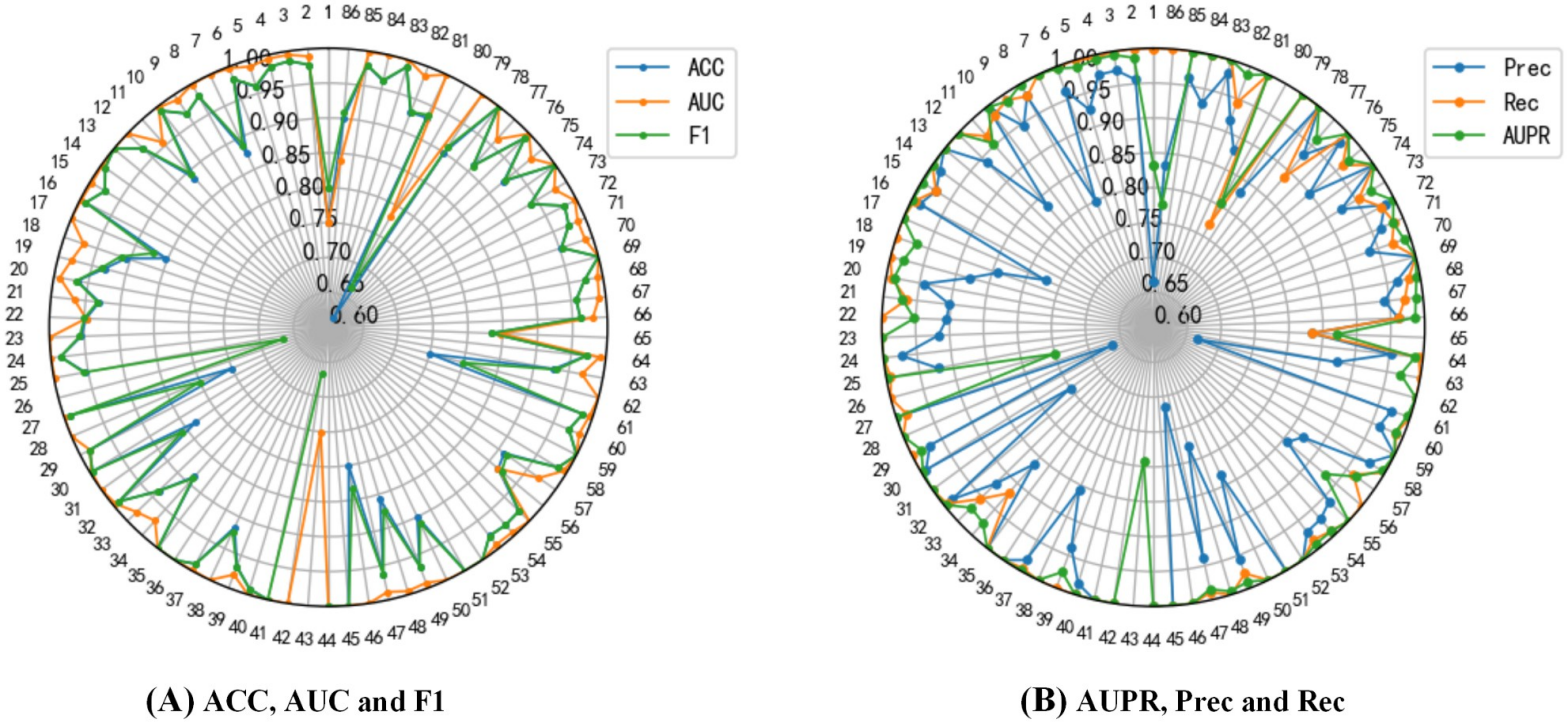
Performance for each DDI type.

Furthermore, we investigated the performances of DGNN-DDI for each DDI type and calculated the metric scores for interaction types independently by using predicted scores and real labels. The performance metrics for each DDI type are shown in Fig 9. Among 86 DDI types, DGNN-DDI achieves the highest AUC scores and the highest AUPR scores in 80 DDI types (more than 85%). In general, Fig 9 demonstrates that DGNN-DDI produces good performance in most of DDI types.

### Case study

The emergence of severe acute respiratory syndrome coronavirus 2 (SARS-CoV-2) in 2019 has triggered an ongoing global pandemic of the severe pneumonia-like disease coronavirus disease 2019 (COVID-19) [30]. However, the research and development of traditional medicines for the new coronavirus are very expensive in terms of time, manpower, and funds. We hypothesized that combining drugs with independent mechanisms of action could result in synergy against SARS-CoV-2, thus generating better antiviral efficacy [45]. We prioritized 73 combinations of 32 drugs with potential activity against SARS-CoV-2 and then tested them [46]. Twelve synergistic combination drugs were identified. To further investigate which substructure among the 12 synergistic instances contributes most significantly to medication synergistic combos, we visualized the most crucial substructures for combination drugs. Specifically, we first determine the indices (*h*, *t*) of the highest pairwise interaction score from Eq 1:

$$(h,t)=\text{arg}{\text{max}}_{i,j}({\hat{g}}_{x}^{\left(i\right)}M_{r}{\hat{g}}_{y}^{\left(j\right)})\;i,j=1,\ldots ,L$$ (1)

This can be extended to top *k* pairwise interaction scores for further analysis. To keep the study simple, we used only the highest one(*k* = 1). (*h*, *t*) tells us that the substructures of concern are from the *h*-th Multi-GNN layer for *d*_*x*_ and *t*-th Multi-GNN layer for *d*_*y*_, which were primarily responsible for the DDI outcome. We chose three atoms with the largest substructures attention, which were described by Eq 13, as the center of the most vital substructures.

**Fig 10:**
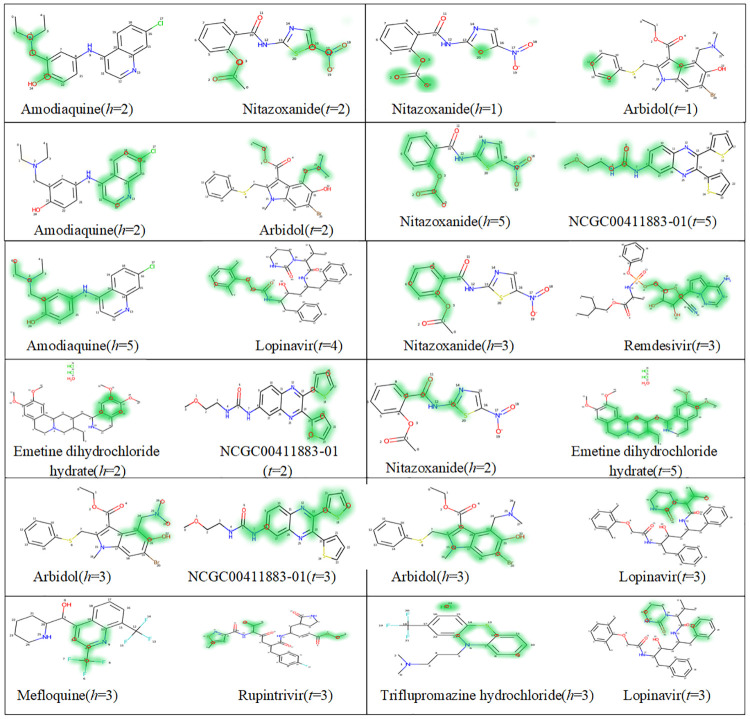
The key substructures contributing to the SARS-CoV-2 drug combinations. The center of the most important substructure and its receptive field are shown as red circle and green colors respectively.

**Fig 11:**
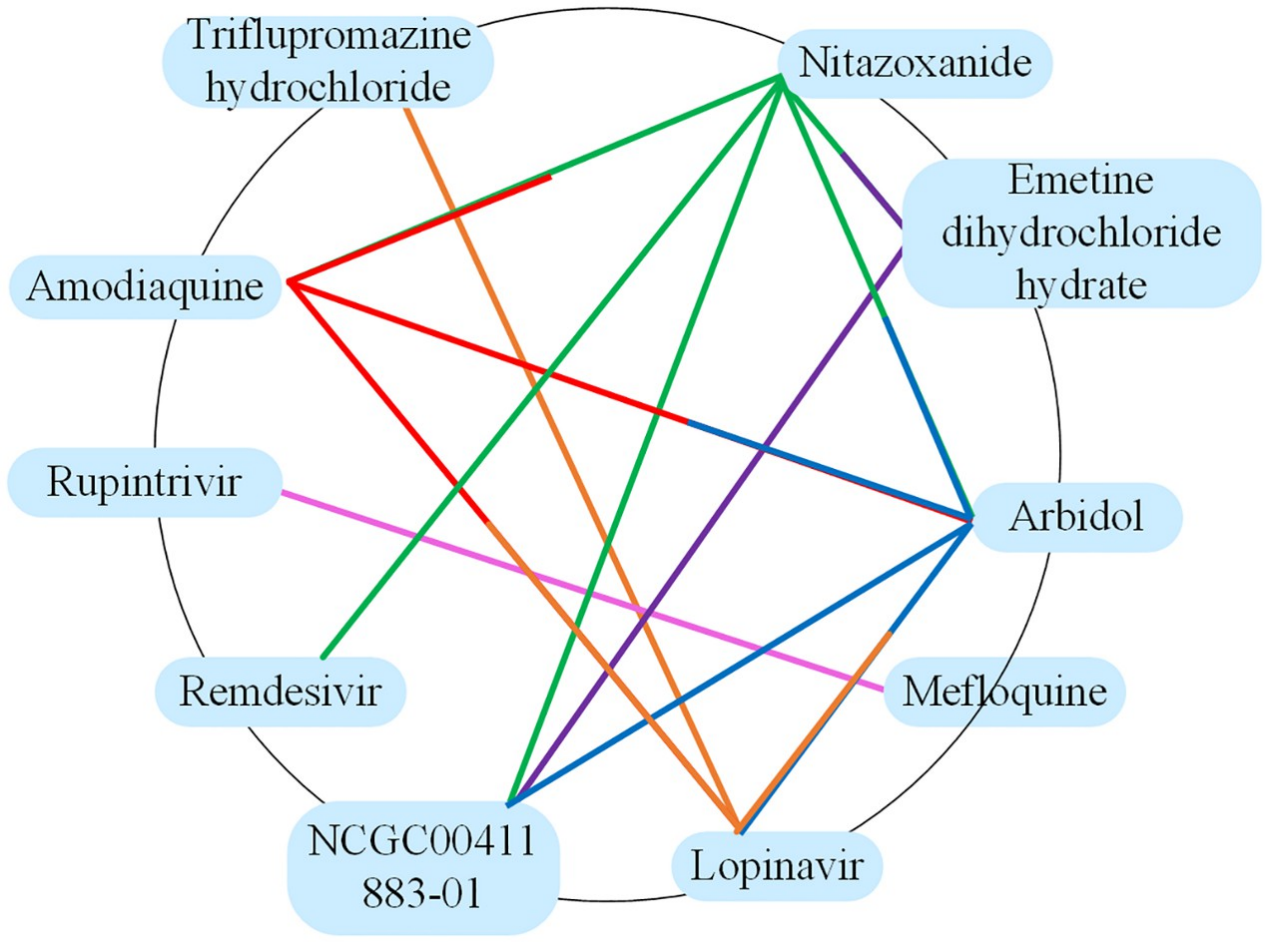
Drug combination to treat SARS-CoV-2.

Fig 10 show the results of this case study. Contributions of substructures are presented as a heat map (map with green fill) of the molecular graph. Each row contains two pair of drugs, for each pair of drugs, the indices *h*, *t* means the radius of a substructures. Therefore, in the heat map, each substructure contribution is shown mainly concentrated around its center. We can see that the drug nitazoxanide with remdesivir, amodiaquine, emetine dihydrochloride hydrate, arbidol and NCGC00411883-01 exhibiting significant synergy against SARS-CoV-2, which is consistent with the result of a previous study [46]. When synergistic with different drugs, the key substructures of drug nitazoxanide are basically the unified, and cresyl acetate(‘CC (= O)Oc1ccccc1C’) or part of it can be found in all of them. However, the key substructures of drug arbidol are vary wildly. We hypothesized that this variety might be caused by various chemical substructures that function in various ways in the medication combinations used to treat SARS-CoV-2, which was consistent with the notion put out that substructures with various weights affect DDI prediction. Overall, these results highlight the utility of drug repurposing and preclinical testing of drug combinations for discovering potential therapies to treat SARS-CoV-2. Additionally, Fig 11 displays a map of pharmacological combinations with results of their synergism.

## Conclusion

This paper presented a novel molecular structure-based deep learning model DGNN-DDI for predicting DDIs between a pair of drugs. The DGNN-DDI used the substructure attention and co-attention mechanism to obtain the substructure with irregular size and shape, and enhance the representation capability for the model. On DrugBank dataset, we contrasted the suggested model with cutting-edge approaches to confirm its superiority. Moreover, we visualized the atom similarity of certain molecules. Finally, we showed the key substructures for the SARS-CoV-2 drug combinations as a case study. The visual interpretation results showed that the DGNN-DDI was sensitive to the structure information of drugs and able to detect the key substructures for DDIs. These advantages demonstrated that the proposed method improved the performance and interpretation capability of DDIs prediction modeling.

## Materials and method

**Fig 12:**
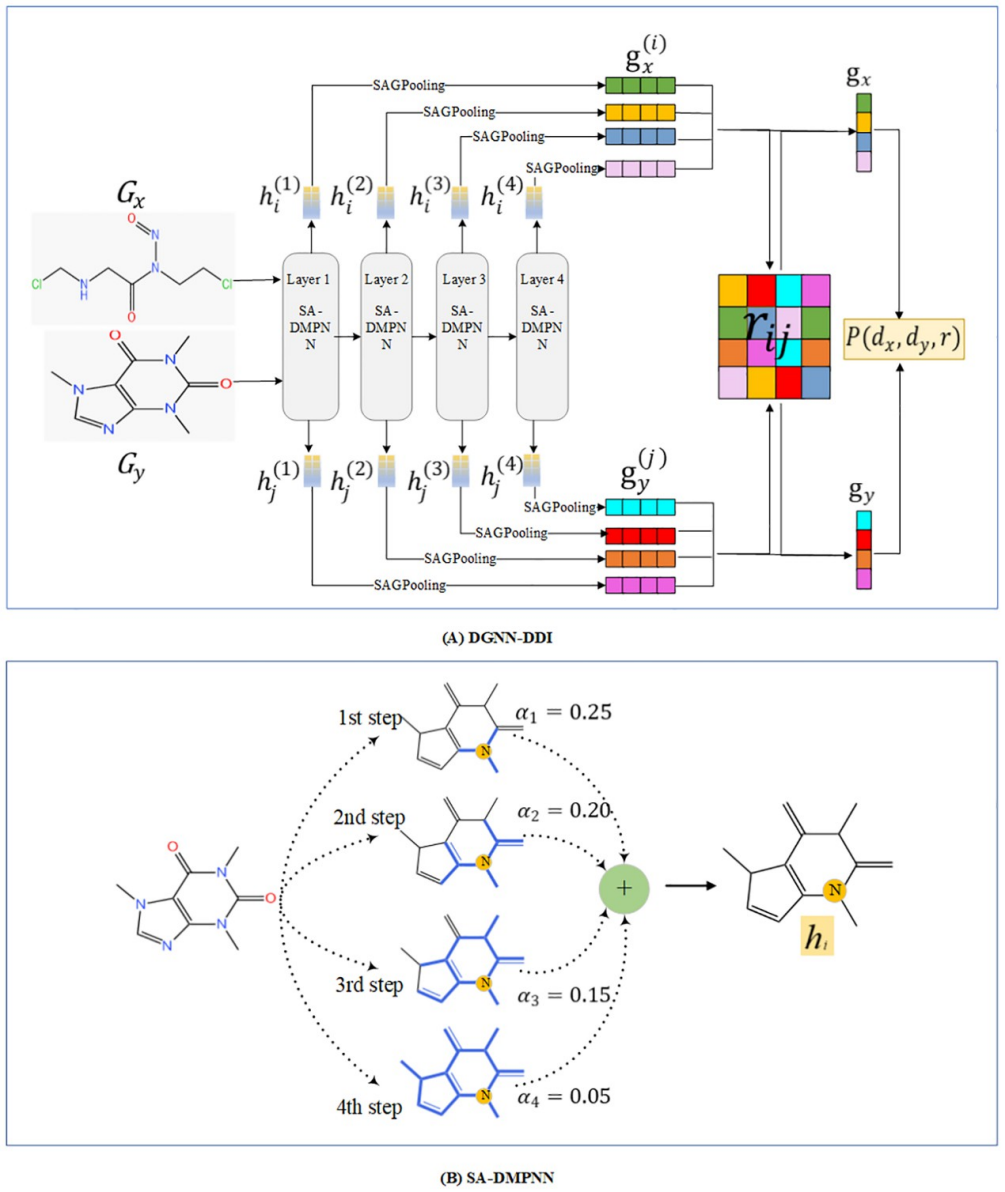
The overview of proposed DGNN-DDI for DDI prediction. (A) The workflow of DGNN-DDI. (B) The SA-DMPNN updates the node-level features with *T* steps where *T* is 4 in this example.

This section gives the technical details of the DGNN-DDI. First, we defined the problem that has to be resolved. Then, we presented the input representation and all involved computational steps of our method. The overall framework is shown in Fig 12.

### Problem formulation

The purpose of the DDIs prediction task is to develop an advanced model that takes two drugs and an interaction type as input and generates an output indicating whether there exists an interaction between them. Formally, given a dataset of DDIs ${\left.M=\{{\left(d_{x},d_{y},r\right)}_{i}\}\right|}_{i=1}^{N}$, where *d*_*x*_, *d*_*y*_ is taken from the drugs set *D*, *r* denotes the interaction type between two drugs, taken from the interaction types set *I*. Our major objective is to find a model *f*: *D* × *D* × *I* → {0, 1}, which predicts the probability that this type of interaction will occur between the two drugs.

### Input representation

**Fig 13:**
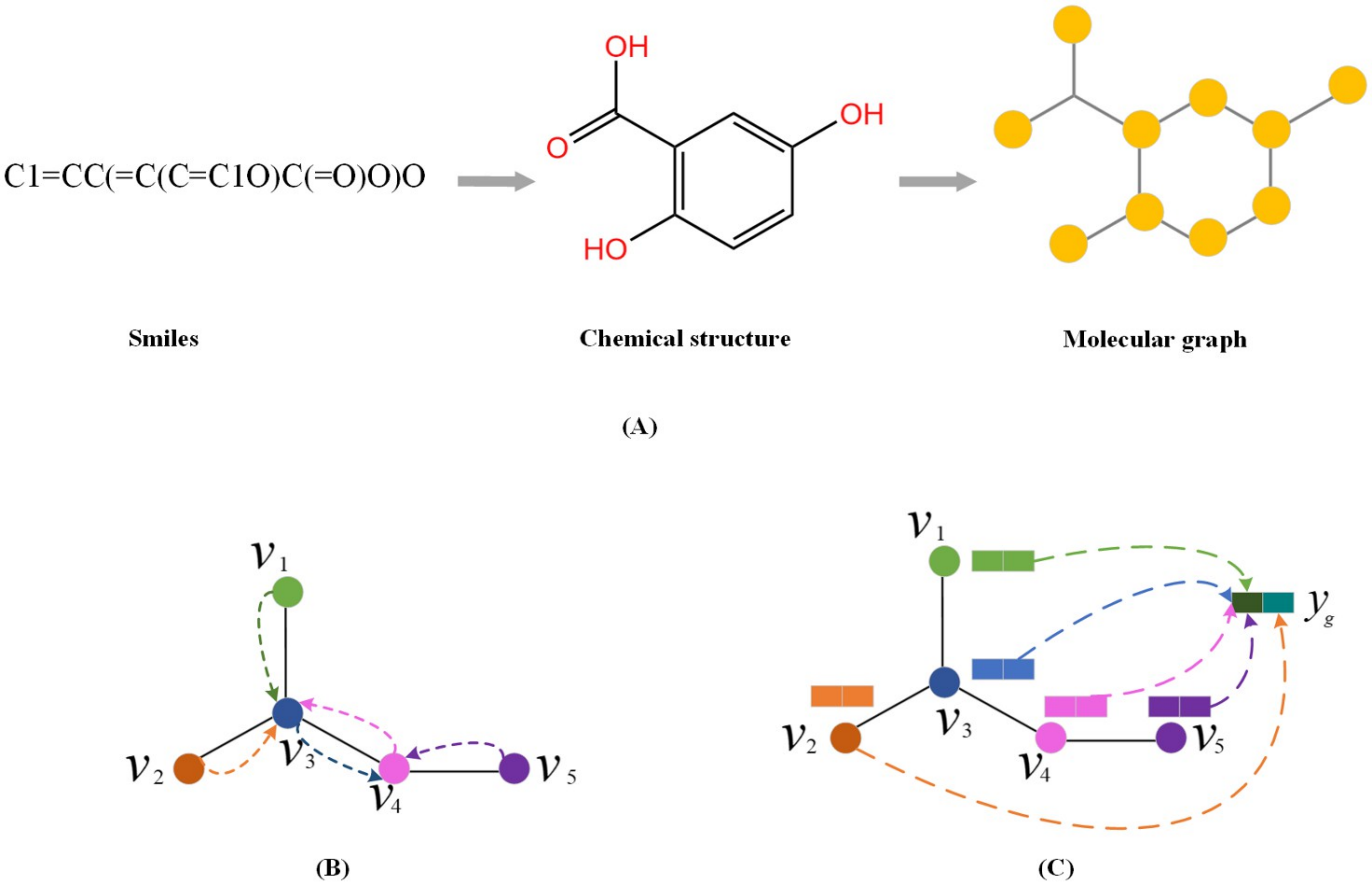
Molecule representation and graph embedding. (A) Preprocessed the smiles into graph. (B) Graph message passing phase. (C) Graph readout phase.

The input of the model is a DDI tuple (*d*_*x*_, *d*_*y*_, *r*). Drugs *d*_*x*_ and *d*_*y*_ are both represented by SMILES strings. We preprocessed the SMILES into graph using RDKit [47] as shown in Fig 13A, where the nodes representing atoms, while edges representing the bonds between the atoms. Therefore, a drug is typically defined as a molecular graph *G* = (*V*, *E*), where $V=\left.{\left\{v_{i}\right\}}_{i=1}^{n}\right|$ is the set of nodes, and $E={\left.\left\{{\left(v_{i},v_{j}\right)}_{s}\right\}\right|}_{s=1}^{m}$ is the set of edges. Each node *v*_*i*_ has a corresponding feature vector *x*_*i*_ ∈ *R*^*d*^. Similarly, while each edge *e*_*ij*_ = (*v*_*i*_, *v*_*j*_) has a feature vector *x*_*ij*_ ∈ *R*^*d*′^. The features used for atoms and bonds are given in the Table 3.

**Table 3:** Atom and bond features.

atom feature	Description	Size
atom type	[B, C, N, O, F, Si, P, S, Cl, As, Se, Br, Te, I, At, meta	16 (one-hot)
degree	number of covalent bonds [0, 1, 2, 3, 4, 5, 6, 7, 8, 9, 10]	11 (one-hot)
hybridization	[sp, sp2, sp3, sp3d, sp3d2]	5 (one-hot)
implicit valence	implicit valence of the atom [0, 1, 2, 3, 4, 5, 6,]	7 (one-hot)
radical electrons	number of radical electrons	1(integer)
formal charge	formal charge of the atom	1 (integer)
aromatic	whether the atom is part of an aromatic system	1 (integer)
bond feature	Description	Size
bone type	[single, double, triple, aromatic]	4 (one-hot)
conjugated	whether the bond is part of a conjugated system	1 (integer)
ring	whether the bond is part of a ring	1 (integer)

### Graph neural network

When a graph is represented as *G* = (*V*, *E*), a GNN maps a graph *G* to a vector *h*_*G*_ ∈ *R*^*d*^ usually with a message passing phase and readout phase. As shown in Fig 13B and 13C, the message passing phase updates node-level features by aggregating messages from their neighbor nodes in *G*, and the readout phase generates a graph-level feature vector by aggregating all the node-level features, which is used to predict the label of the graph.

#### Message passing phase

Given a graph *G*, we denoted the feature of node *v* at step *t* as $x_{v}^{\left(t\right)}\in R^{d}$. We then updated $x_{v}^{\left(t\right)}$ into $x_{v}^{\left(t+1\right)}\in R^{d}$ using the following graph convolutional layer [9]:

$$m_{v}^{\left(t+1\right)}=\sum_{u\in N(v)}M_{t}(x_{v}^{\left(t\right)},x_{u}^{\left(t\right)},e_{uv})$$ (2)

$$x_{v}^{\left(t+1\right)}=U_{t}(x_{v}^{\left(t\right)},m_{v}^{\left(t+1\right)})$$ (3)

where *N*(*v*) denotes the neighbors of *v* in graph *G*. *M*_*t*_ and *U*_*t*_ are the message functions and node update functions, respectively.

#### Readout phase

To obtain a graph-level feature *h*_*G*_, readout operation integrates all the node features among the graph *G* is given in Eq 4:

$$h_{G}=R(x_{v}^{T}:v\in G)$$ (4)

where *R* is readout function, and *T* is the final step.

**Fig 14:**
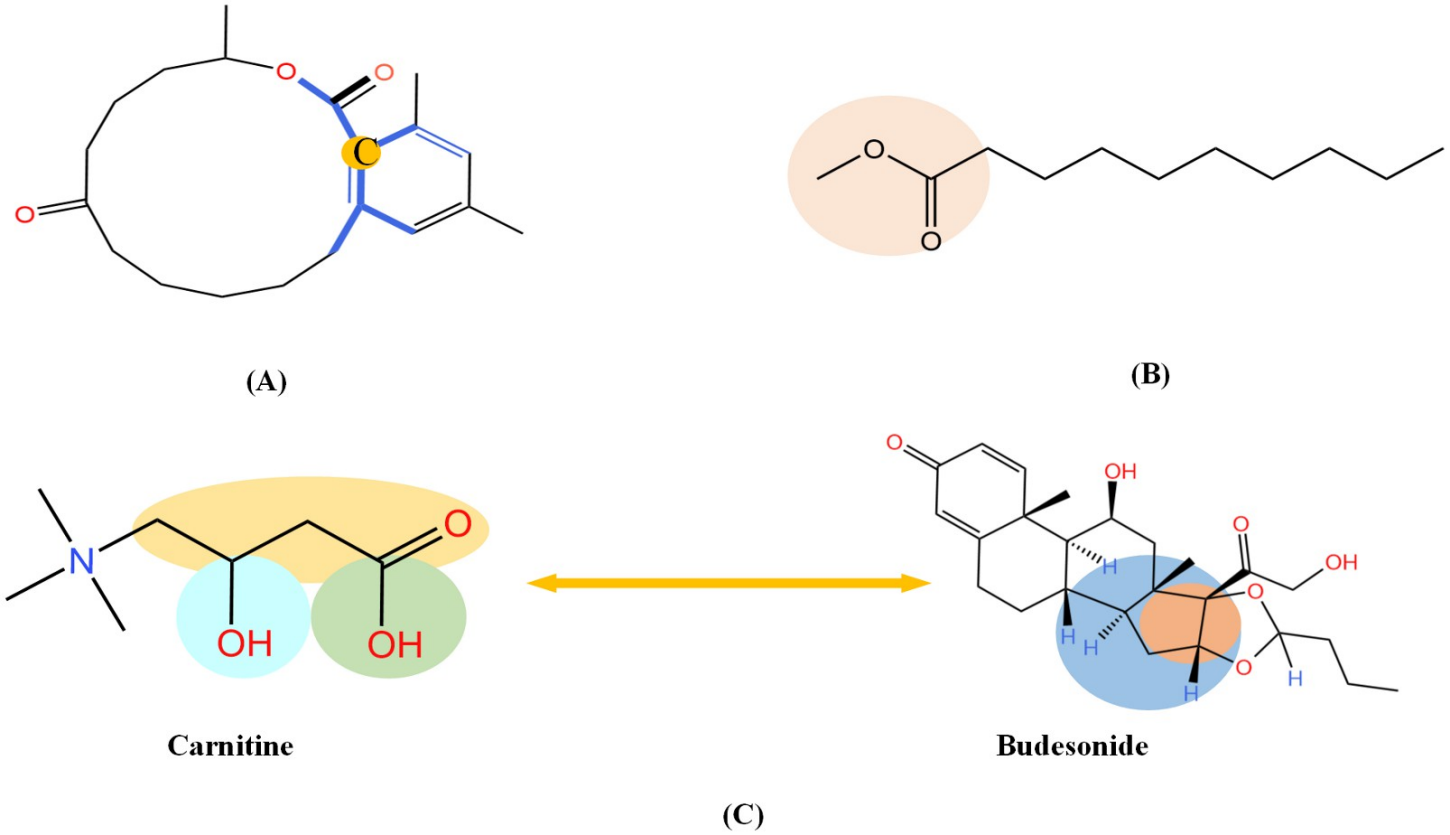
Both structure information and DDIs are important for GNN. (A) The sight of GNNs in the second layer is shown in blue as we take the carbon with orange as the center. In this example, a GNN with two layers fails to identify the ring structure of zearalenone. (B) The GNN should preserve local structure information (orange ellipse) (C) The interaction type of ‘blood calcium increased’ between drug pair ‘Carnitine’ and ‘Budesonide’ is caused by their partial significant substructures (elliptical parts).

So far, the GNN is learned in a standard manner, which has third shortcomings for DDIs prediction. First, the GNN extracts fixed-size substructures with a predetermined number of layers, it is insufficient to capture the global structure of the molecules. As shown in Fig 14A, a GNN with two layers is unable to know whether the ring exists in the molecule. Therefore, to capture the structures make up of *k*-hop neighbors, the *k* graph convolutional layers should be stacked. Second, a well-constructed GNN should be able to preserve the local structure of a compound. As shown in Fig 14B, the methyl carboxylate moiety is crucial for methyl decanoate and the GNN should distinguish it from the less essential substituents in order to make a reasonable inference. Concretely, it is necessary to apply the attention mechanism to the key substructures. Third, DDIs usually depend only on a few substructures of the whole chemical structures. As depicted in Fig 14C, the interaction type of ‘blood calcium increased’ between drug pair ‘Carnitine’ and ‘Budesonide’ is caused by their partial important substructures [48]. It is feasible to break down DDIs into substructure–substructure interactions. The following, we adopted directed message passing neural network with substructure attention mechanism (SA-DMPNN) and interaction-aware substructure extraction to solve these three limitations.

### Directed message passing neural network with substructure attention mechanism

The idea of substructure attention is to extract substructures with arbitrary sizes and shapes and assign each substructure a unique score. We used the D-MPNN [2] with substructure attention mechanism(SA-DMPNN) for molecule substructures extraction. The process is shown in Fig 12B. During the *t*-th step, the SA-DMPNN extracts substructures with a radius of *t*.

In the SA-DMPNN, each node will receive a message from the bond-level hidden feature. For each node *v*_*i*_, the hidden feature at step *t* is $h_{i}^{\left(t\right)}\in R^{d}$, where $h_{i}^{\left(0\right)}=x_{i}$, we used $h_{ij}^{\left(t\right)}$ to represent a bond-level hidden feature with each bond *e*_*i*→*j*_. We initialized the bond-level hidden features as

$$h_{ij}^{\left(0\right)}=W_{i}x_{i}+W_{j}x_{j}+W_{ij}x_{ij}$$ (5)

where *W*_*i*_ ∈ *R*^*h*×*d*^, *W*_*j*_ ∈ *R*^*h*×*d*^, and *W*_*ij*_ ∈ *R*^*h*×*d*′^ are learnable weight matrices.

At step *t*, we computed the bond-level neighborhood features for each node before utilizing a substructure-aware global pooling, then we obtained its bond -level graph representation *g*^(*t*)^. The corresponding calculation equations are thus:

$$m_{i}^{\left(t\right)}=\sum_{v_{j}\in N(v_{i})}\beta_{ij}h_{ji}^{\left(t\right)}$$ (6)

$$g^{\left(t\right)}=\sum_{i=1}^{n}m_{i}^{\left(t\right)}$$ (7)

where SAGPooling [49] can be used to calculate *β*_*ij*_. Given a graph with bond-level feature matrix *X* and adjacency matrix *A* in which the nonzero position indicates that two bonds share a common node, SAGPooling computes the importance vector *β*_*ij*_ as follows:

$$\beta_{ij}=\text{softmax}(GNN(A,X))$$ (8)

GNN is an arbitrary GNN layer for calculating projection scores. For each bond-level graph representation *g*^(*t*)^, the substructure attention score can be computed as follows:

$$e^{\left(t\right)}=w^{\left(t\right)}⊙\text{tanh}(Wg^{\left(t\right)}+b)$$ (9)

where ⊙ represents dot product, *w*^(*t*)^ is a weight vector for step *t*. In order to make the coefficients of different steps easy to compare, we normalized *e*^(*t*)^ by using the softmax function:

$$\alpha^{\left(t\right)}=\frac{\text{exp}(e^{\left(t\right)})}{\sum_{k=1}^{T}\text{exp}(e^{\left(k\right)})}$$ (10)

where each *α*^(*t*)^ ∈ *R*^1^ indicates the importance of the substructures with a radius of *t*. After updating bond-level hidden features *T* steps, we returned the final representation of *h*_*ij*_ by the weighted sum of bond-level hidden features across all steps according to the following:

$$h_{ji}=\sum_{t=1}^{T}\alpha^{\left(t\right)}h_{ji}^{\left(t\right)}$$ (11)

The substructure attention mechanism will make it possible that not all the nodes in a substructure are considered equally, refining even further the type of substructures being learned.

Finally, we returned to the node-level features by aggregating the incoming bond-level features as follows:

$$h_{i}=f(x_{i}+\sum_{v_{j}\in N(v_{i})}h_{ji})$$ (12)

where *f* is a multilayer perceptron, and *h*_*i*_ contains the substructure information from different receptive fields centering at *i*-th atom.

### Interaction-aware substructure extraction

As mentioned above, shallow convolutional layers cannot capture global structure of the molecules, in order to overcome this limitation, we stacked multiple SA-DMPNN blocks to obtain substructure-level graph representation. The stacking structure is referred to as Multi-GNN for the sake of simplicity in descriptions. The process is shown in Fig 12A.

For a given drug *d*_*x*_, suppose we have obtained the node-level features for each node in molecular graph *G*_*x*_ from the SA-DMPNN. At each Multi-GNN layer *l*, the features of each node are denoted as $h_{i}^{\left(l\right)}$, then the representation of the substructure $g_{x}^{\left(l\right)}\in G_{x}$ is therefore given by the Eq 13, which is represented by aggregating the node features $h_{i}^{\left(l\right)}$, each one weighted by a learnable coefficient *β*_*i*_, which can be interpreted as its importance. The *β*_*i*_ can be obtained by the SAGPooling.


$$g_{x}^{\left(l\right)}=\sum_{i=1}^{n}\beta_{i}h_{i}^{\left(l\right)}$$ (13)

After obtaining all the substructure information $g_{x}^{\left(l\right)}$ and $g_{y}^{\left(l\right)}$ of the input drugs *d*_*x*_ and *d*_*y*_ from all the Multi-GNN layers, we employed a co-attention mechanism to account for the importance *γ*_*ij*_ of each pairwise interaction between the substructures of *G*_*x*_ and *G*_*y*_, which is given by:

$$\gamma_{ij}=b^{T}\;\text{tanh}(W_{x}g_{x}^{\left(i\right)}+W_{y}g_{y}^{\left(j\right)})\;i,j=1,\ldots ,L$$ (14)

where *b* is a learnable weight vector, *W*_*x*_ and *W*_*y*_ are learnable weight matrices. To prevent situations where similar substructures are given high ratings, we applied various weight matrices.

Furthermore, we updated the substructural features $g_{x}^{\left(i\right)},g_{y}^{\left(j\right)}$ with *γ*_*ij*_, respectively, which is formulated as follows:

$${\hat{g}}_{x}^{\left(i\right)}=\sum_{j=1}^{L}\gamma_{ij}g_{x}^{\left(i\right)}\;i=1,\ldots ,L$$ (15)

$${\hat{g}}_{y}^{\left(j\right)}=\sum_{i=1}^{L}\gamma_{ij}g_{y}^{\left(j\right)}\;j=1,\ldots ,L$$ (16)

Finally, the graph-level representation of *d*_*x*_ can be computed by the following:

$$g_{x}=\sum_{l=1}^{L}{\hat{g}}_{x}^{\left(l\right)}$$ (17)

**Fig 15:**
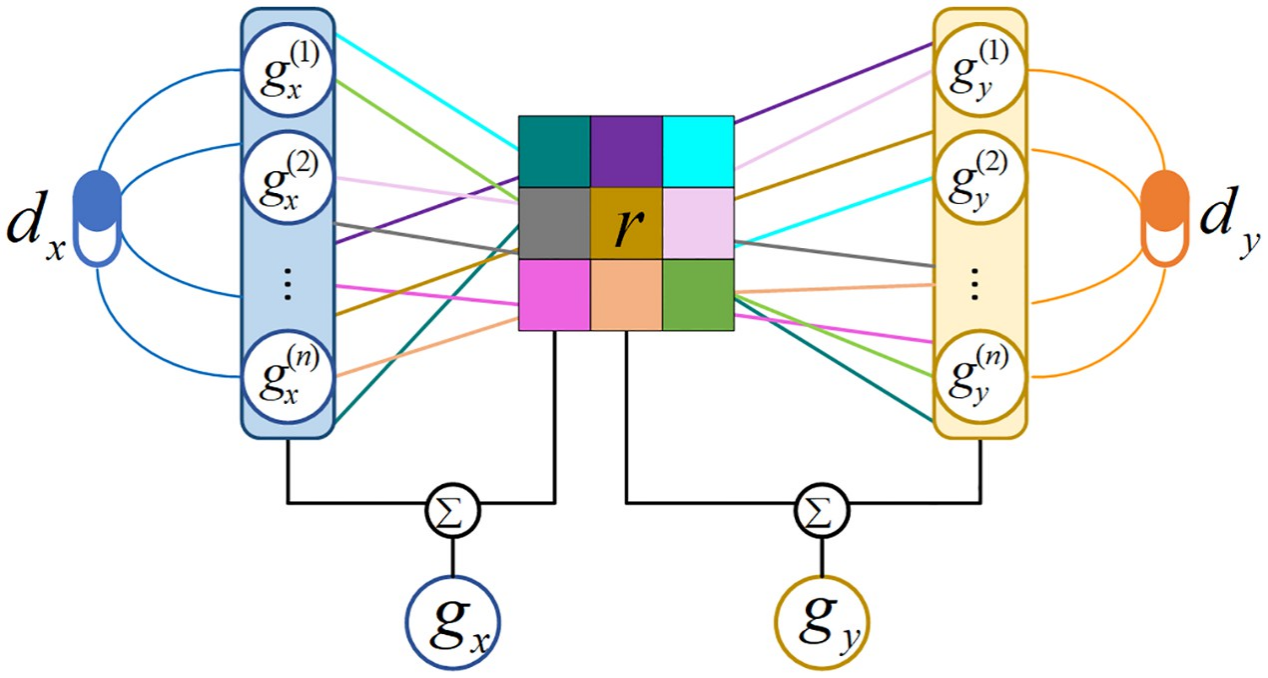
The overall computational steps for graph-level representation of *d*_*x*_ and *d*_*y*_.

The graph-level representation of *d*_*y*_(i.e., *g*_*y*_) can be calculated by using computational steps similar to that described in Eq 17. As opposed to the global pooling that considers every substructure equally important, we utilized the interaction information to enhance structure information of *d*_*x*_ and *d*_*y*_ by assigning cross-substructure interaction scores. The overall computational steps for graph-level representation of *d*_*x*_ and *d*_*y*_ are depicted in Fig 15.

### Drug-drug interaction prediction

Given a DDI tuple (*d*_*x*_, *d*_*y*_, *r*), the DDIs prediction can be expressed as the join probability of the tuple:

$$P(d_{x},d_{y},r)=\sigma (g_{x}^{T}M_{r}g_{y})$$ (18)

where *σ* is the sigmoid function, and *M*_*r*_ is the learnable matrix representation of interaction type *r*. The learning process of the model can be achieved by minimizing the cross-entropy loss function [50], which is given as follows:

$$Loss=-\frac{1}{M}\sum_{i=1}^{M}y_{i}\;\text{log}(p_{i})+(1-y_{i})\text{log}(1-p_{i})$$ (19)

where *y*_*i*_ = 1 indicates that an interaction exists between *d*_*x*_ and *d*_*y*_, and vice versa; and *p*_*i*_ is the predictive interaction probability of a DDI tuple is computed by using Eq 18.
